# Three-year migration analysis of a new metaphyseal anchoring short femoral stem in THA using EBRA-FCA

**DOI:** 10.1038/s41598-022-22160-w

**Published:** 2022-10-13

**Authors:** Patrick Reinbacher, Maria Anna Smolle, Joerg Friesenbichler, Alexander Draschl, Andreas Leithner, Werner Maurer-Ertl

**Affiliations:** Department of Orthopaedics and Trauma, Medical University of Graz, Auenbruggerplatz 5, 8036 Graz, Austria

**Keywords:** Medical research, Musculoskeletal system

## Abstract

Cementless calcar-guided femoral short stems in total hip arthroplasty (THA) have become increasingly popular over the years. Early distal migration of femoral stems measured by Einzel-Bild-Roentgen Analyse, Femoral Component Analyse (EBRA-FCA) has been reported to be a risk factor for aseptic loosening. The aim of this study was to analyse axial migration behavior and subsidence of a new short stem (launched in 2015) over a follow-up period of 3 years. According to the study protocol, 100 hip osteoarthritis patients who consecutively received an unilateral cementless calcar-guided short stem (ANA.NOVA proxy) at a single department were prospectively included in this mid-term follow-up study. Thirteen patients were lost to follow-up, resulting in 87 patients with unilateral THA who fulfilled the criteria for migration analysis with EBRA-FCA. The cohort comprised 41 males (mean age: 60 ± 16.5; mean BMI (Body Mass Index): 30 ± 13) and 46 females (mean age: 61 ± 15.5; mean BMI: 27 ± 10). Seven standardized radiographs per patient were analyzed with EBRA-FCA. An average migration of 2.0 mm (0.95–3.35) was observed within the first 3 years. The median increase during the first year was higher than in the second and third year (1.2 mm [IQR: 0.5–2.15] vs. 0.3 mm [IQR: 0.1–0.6 mm] vs. 0.25 mm [IQR: 0.1–0.5 mm]. Detected migration did not lead to stem loosening, instability, dislocation, or revision surgery in any patient. A higher risk for subsidence was observed in male and heavyweight patients, whereas the female gender was associated with a lower risk. No correlation between migration and revision could be observed. Although moderate subsidence was detectable, the performance of the short stem ANA.NOVA proxy is encouraging. Yet, its use may be re-considered in overweight and male patients due to more pronounced subsidence.

## Introduction

In the last decades, the number of primary total hip arthroplasty (THA) has increased steadily^1,2^, and indications for THA have expanded to younger and more active patients^3–7^. Along with it, a rising number of revision THA has been observed, especially in patients under the age of 65 years who are known to be at higher risk for surgical revision in the future^8–10^. Femoral short stems were first introduced in 1989^11^, and their use in THA has become increasingly popular over the last two decades^12–15^. In addition, due to technical developments, various bone-preserving cementless metaphyseal-anchoring femoral short stem designs have emerged^12,15^.

Short-stemmed femoral implants were designed to preserve metaphyseal and diaphyseal bone stock through proximal load transfer, allowing revision surgery with conventional standard-length stems^12,14–16^. Compared with conventional uncemented straight stems, several potential advantages of cementless short stems have been reported in the literature, including more physiological loading in the proximal femur, eventually reducing stress shielding and the incidence of thigh pain, as well as the possibility for minimally invasive implantation techniques^17–19^. Furthermore, the concept of calcar-guided short stems in THA allows for better reconstruction of the individual hip anatomy by following the calcar of the femoral neck^20,21^.

Owing to their design, femoral short stems are reduced in length and diaphyseal fixation compared to conventional stems^12,20,22,23^. This has raised concerns regarding their primary and secondary stability and, consequently, implant survival^20,24–27^. Furthermore, a tendency towards early distal migration (axial subsidence) has been observed in short stem THAs that might be caused by their mainly metaphyseal anchorage and smaller bone-implant interface^20,24–27^. With primary stability being a prerequisite for bony ingrowth, the amount of distal migration detected with EBRA-FCA (Einzel-Bild-Roentgen Analyse, Femoral Component Analyse) has shown to be a good predictive indicator for aseptic loosening in conventional stems, whereby distal subsidence of more than 1.5 mm or 2.7 mm within 2 years postoperatively is considered a risk factor for early loosening^28–31^.

Although radiostereometric analysis (RSA) is considered the gold standard for migration analysis, EBRA-FCA allows accurate distal migration measurement, offering a valid tool for detecting axial subsidence in a non-invasive manner and applicability in a retrospective study design^31–33^. However, it remains unclear if the suggested EBRA-FCA thresholds that have been developed for straight stems^29,31^ can be transferred to short stems, considering that many short stem designs result in different bone remodeling and migration patterns^34^. While conventional stems usually offer initial stability followed by secondary subsidence, short stems rather show early migration followed by secondary stabilization, also known as the “settling effect”^25,26,35,36^. This effect may indicate, despite early migration, a proper secondary fixation in short stems and thus long durability^33^. Supporting this assumption, a recent study has reported comparable long-term survival of short and conventional stems with no additional risk of aseptic loosening in short stems^37^. Furthermore, no significant difference in cumulative probability of revision 5 years after THA was found between modern calcar-guided short stems and conventional stems^38^. However, there is a lack of data confirming long-term durability of the latest generation of short stems, despite promising short- and mid-term results^32,36,39,40^.

Given the current knowledge, there is a need to determine the clinical relevance of short stems’ early subsidence and whether this phenomenon is followed by secondary fixation with long durability. Although biomechanics of modern calcar-guided short stems are not fully understood, it is not surprising that the rate of subsidence is associated with the stem design^25,27^.

Therefore, the current study aimed at investigating the migration behavior of a novel, metaphyseal-anchoring, calcar-guided, neck-sparing short-stem (ANA.NOVA proxy; ImplanTec, Moedling, Austria) for the first time in this 3-year follow-up study. According to van Oldenrijk et al.^12^, this short stem can be classified as “partial collum”. As our study follows a retrospective design, EBRA-FCA was performed to detect axial migration of the femoral short stem. Furthermore, we evaluated the possible influence of age, gender, BMI, Dorr type, and CCD on stem subsidence.

## Materials and methods

The herein used stem (ANA.NOVA proxy (ImplanTec, Moedling, Austria)), was launched onto the market in Austria in 2015. It is designed for cementless, calcar-guided, press-fit application with a 3-point anchorage. The surface consists of biocompatible titanium alloy and a rough titanium plasma coating. Further support of osteointegration can be achieved with electrochemically applied BONIT, mainly consisting of nanocristallyne hydroxyapatite. Stem fixation takes place epiphyseally at the femoral neck, metaphyseally proximal (between the medial calcar and lateral cortex), and tapered in the distal meta-diaphysis. Due to its triple-tapered, trapezoidal design, the main fixation zone is between the medial calcar and the lateral cortex. Sizes range from 0 to 11, with two offset options (standard and lateral offset, although only the standard offset was available at time of the study). According to the classification of van Oldenrijk et al.^12^, it is a partial femoral neck-sparing short stem.

One hundred primary hip osteoarthritis patients who consecutively underwent elective total hip arthroplasty at a single institution, were prospectively enrolled in the study. Based on x-rays, femoral short stem sizes were planned preoperatively with MediCAD 2D (mediCAD, Hectec GmbH, Altdorf, Germany) as described in a previously conducted study^41^. In all patients, a single surgeon implanted the same short stem with an anterolateral approach between February 2016 and July 2017.

The following inclusion criteria were applied: primary hip osteoarthritis as an indication for THA, availability of preoperative radiograph, a series of at least three consecutive standardized radiographs accepted by the EBRA-FCA software (University of Innsbruck, Austria), and the acceptance of taking radiographs immediately after surgery and during 3 years of follow-up.

Of 100 prospectively included patients, one died unrelatedly to the operation within the first year after surgery. The other 99 patients were consecutively followed up with clinical and radiographic postoperative assessments scheduled 6 weeks, 3 months, 6 months, 12 months, 24 months, and 36 months postoperatively. Each time standardized standing x-rays of the pelvis (a.p.) and hip (a.p. and oblique) were performed.

**Figure 1:**
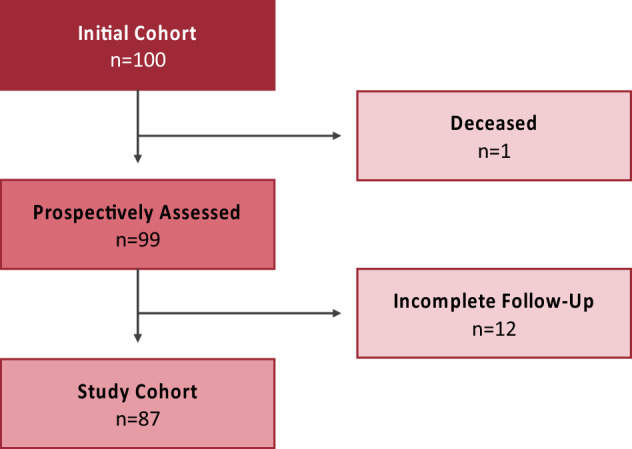
Flow-chart of the study cohort.

Twelve patients were subsequently excluded due to incomplete appointments, resulting in 87 patients with unilateral THA eligible for analysis (Fig. 1).

Postoperative mobilization was carried out by full weight bearing from the day of surgery. All patients had crutches under full weight bearing for 6 weeks. All patients followed the same rehabilitation protocol.

The study followed accepted ethical, scientific, and medical standards and was conducted in compliance with recognized international standards, including the Declaration of Helsinki principles. Informed consent was obtained from all the participants, and the local ethics committee approved the study protocol (EK-Nr. 28-152 ex 15/16).

### EBRA-FCA measurements

**Figure 2:**
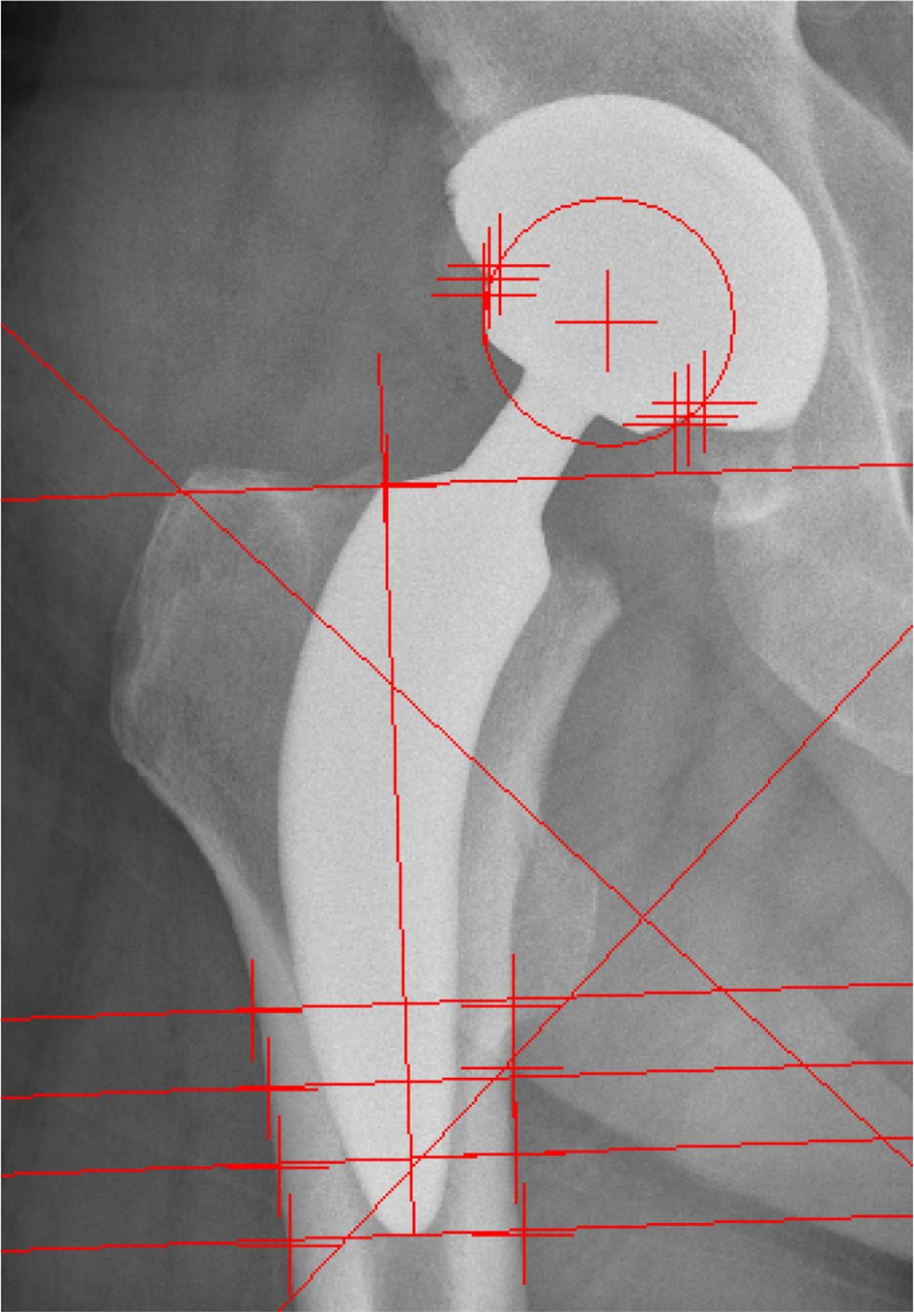
EBRA-FCA.

**Figure 3:**
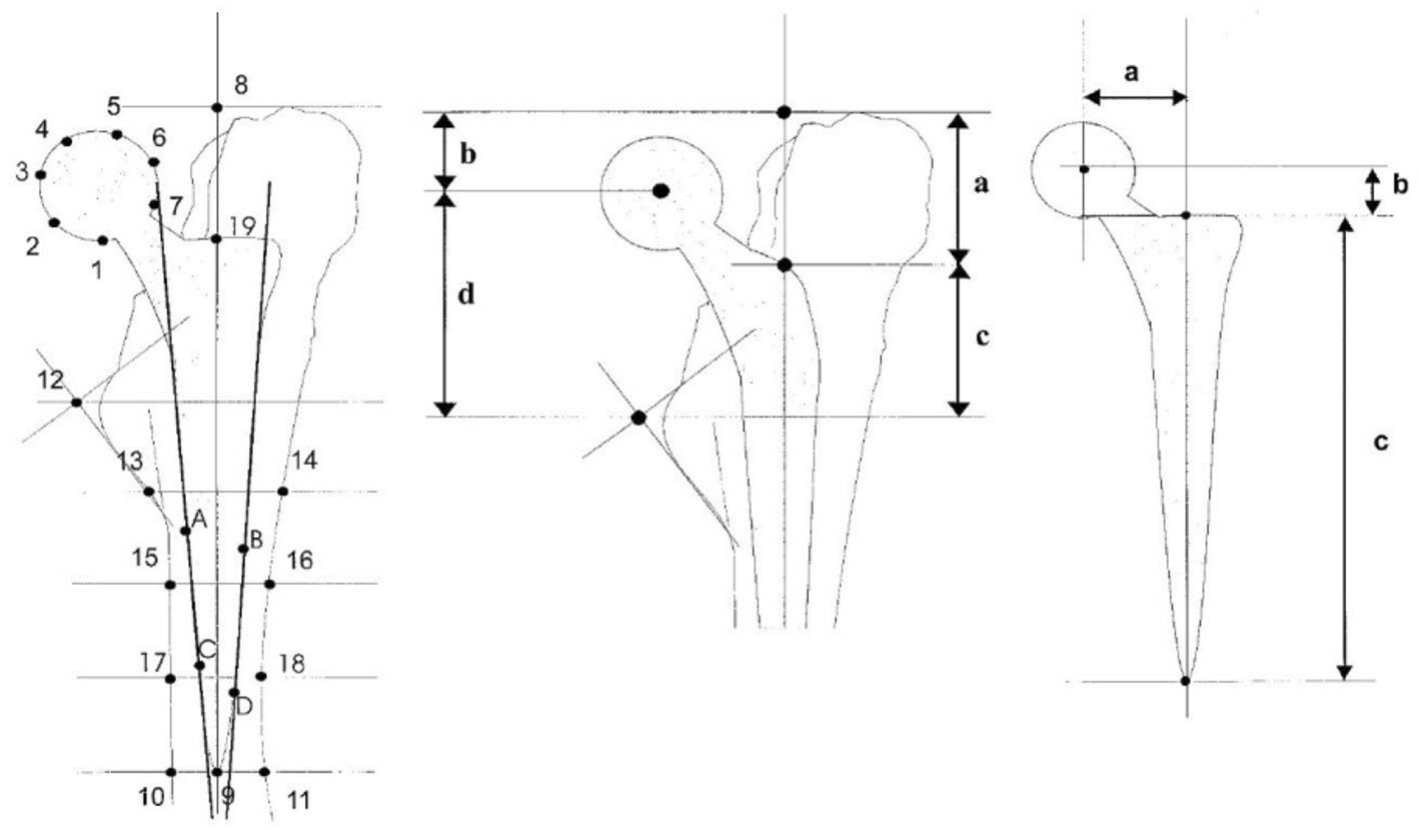
(**a**) Horizontal distance between the center of the prosthetic head and the shoulder point, (**b**) Vertical distance between the center of the prosthetic head and the shoulder point, (**c**) Distance between the shoulder point and the tip of the prosthesis, from R. Biedermann et al. J Bone Joint Surg Br 1999^13^.

Axial femoral stem migration can be determined with EBRA-FCA (Einzel-Bild-Roentgen-Analyse, Femoral Component Analyse)^42^. It is a non-invasive, computer-based measurement method for the migration of implanted stems, which compares digital serial radiographs of the hip. At least three anteroposterior radiographs were obtained from each patient. The measurement was structured as follows (Fig. 2): measurement of the prosthetic head by placing two lines to the lateral and medial side of the stem, setting the so-called “shoulder point”, putting a horizontal line through the trochanter major, positioning two lines to the upper and lower margin of the trochanter minor, placing a horizontal line to the tip of the prosthesis; the final step is to place the “8 pints” at the outmost points of the cortical bone. By this procedure, three parameters can be used for the assessment (Fig. 3): (1) the horizontal distance between the center of the prosthetic head and the shoulder point, (2) the vertical distance between the center of the prosthetic head and the shoulder point, and (3) the distance between the shoulder point and the tip of the prosthesis. According to Biedermann et al.^30^, EBRA-FCA can detect an implant migration of 1 mm with a sensitivity of 78% and a specificity of 100%; the accuracy is stated to be better than ± 1.5 mm^12^.

### Statistical analyses

All statistical analyses were performed with Stata Version 15.1 (StataCorp, College Station, TX 77845 USA). Differences between binary and categorical variables were assessed with Chi-squared tests. T-tests and Wilcoxon-rank-sum-tests were performed to assess differences between normally and non-normally distributed variables, respectively. Interobserver reliability was assessed with the intraclass correlation coefficient. Values < 0.5 indicate poor agreement while values from 0.5 to 0.75 indicate moderate, from 0.75 to 0.9 good, and > 0.9 excellent reliability^43^. Klicken oder tippen Sie hier, um Text einzugeben.Random effects models were used to assess the impact of variables on subsidence over time. A *p* value of < 0.05 was considered statistically significant.

### Ethics approval and consent to participate

The present study has been approved by the Institutional Review Board of the Medical University of Graz, Austria (Ethical Committee No. 28-152 ex 15/16, Chairperson: Prof. J. Haas).

## Results

The study cohort consisted of forty-one male (47.1%) patients with a mean age of 60.9 ± 7.8 years at the time of surgery and 46 female patients (52.1%) with a mean of 61 ± 15.5 years age at the time of surgery (Table 1). The distribution of the implanted short stems based on their size was as follows: size 2 (n = 2, 2.3%), size 3 (n = 7, 8.0%), size 4 (n = 10, 11.5%), size 5 (n = 13, 14.9%), size 6 (n = 15, 17.2%), size 7 (n = 20, 23.0%), size 8 (n = 14, 16.1%), size 9 (n = 6, 6.9%). Further demographic data is visible in Table 1.

**Table 1:** Patient characteristics of the study cohort (n = 87).

Age at surgery (in years; mean ± SD)	60.9 ± 7.8
**Gender**	
Male	41 (47.1%)
Female	46 (52.9%)
BMI (median; IQR)	28.9 [25.2; 31.2]
**Side**	
Left	43 (49.4%)
Right	44 (50.6%)
Length of hospital stay (in days; median; IQR)	7 [6; 8]
**DORR types**	
A	23 (26.4%)
B	57 (65.5%)
C	7 (8.1%)
**ASA**	
1	10 (11.9%)
2	54 (64.3%)
3	18 (21.4%)
4	2 (2.4%)
**Head diameter**	
32 mm	5 (5.8%)
36 mm	82 (94.2%)
Cup size (median; IQR)	54 [52; 56]
Shaft size (median; IQR)	6 [5; 7]
**Total subsidence (in mm; median; IQR)**	
6 weeks	0.25 [0.05; 0.9]
Increase (in mm; median; IQR)	+ 0.2 [0.1; 0.5]
3 months	0.55 [0.2; 1.25]
Increase (in mm; median; IQR)	+ 0.25 [0.1; 0.5]
6 months	0.9 [0.4; 1.8]
Increase (in mm; median; IQR)	+ 0.25 [0.1; 0.5]
12 months	1.2 [0.5; 2.15]
Increase (in mm; median; IQR)	+ 0.3 [0.1; 0.6]
24 months	1.55 [0.7; 2.75]
Increase (in mm; median; IQR)	+ 0.25 [0.1; 0.5]
36 months	2.0 [0.95; 3.35]

*SD* Standard deviation, *BMI* Body mass index, *IQR* Interquartile range.

**Figure 4:**
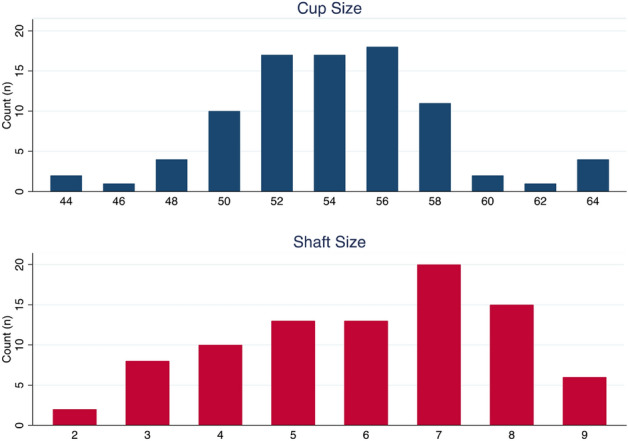
Distribution of cup and shaft sizes of the study cohort.

The most common implanted cup and stem sizes were 56 mm and size 7, respectively (Fig. 4). In 94.2% of cases (n = 82), a 36 mm ceramic head was used. In the remaining 5.8%, a 32 mm ceramic head was implanted. Forty-three THAs (49,4%) were performed on the left and 44 (50.6%) on the right side. The most common Dorr Type in the cohort was Dorr Type B (n = 57 [65.5%], whereas 26.4% (n = 23) had Dorr Type A and 8.1% (n = 7) Dorr Type C. The length of hospital stay was 7 days on average (Table 1).

### Subsidence

**Figure 5:**
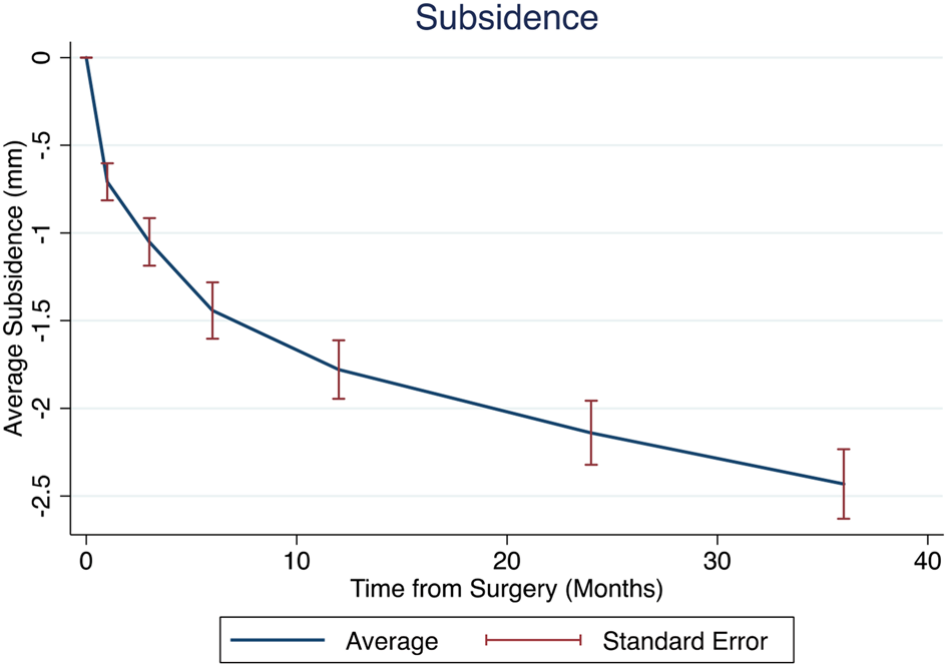
Average subsidence of the study cohort over time.

On the one hand, the axial subsidence of the stem measured with EBRA-FCA increased steadily with average total subsidence of 0.25 mm (IQR: 0.05–0.90 mm) after 6 weeks postoperatively compared to 2.00 mm (0.95–3.35 mm) after 36 months post-surgery (Table 1). On the other hand, the median increase within the first year was higher than in the second and third year (1.20 mm [IQR: 0.5–2.15] vs. 0.30 mm [IQR: 0.10–0.60 mm] vs. 0.25 mm [IQR: 0.10–0.50 mm] (Fig. 5).

### Risk factors for subsidence

**Figure 6:**
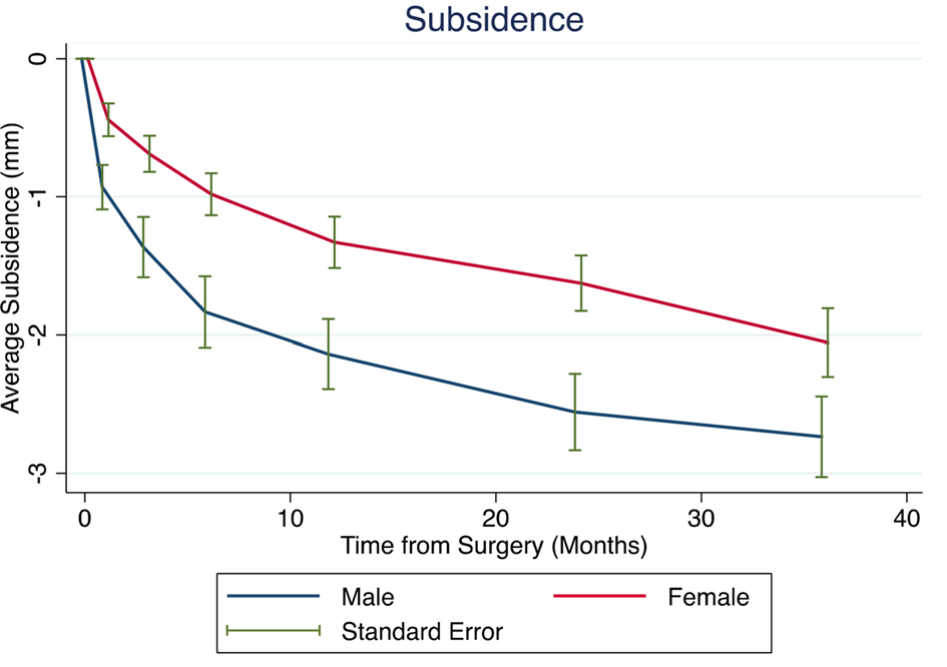
Average subsidence of the study cohort over time separated by gender (red = female; blue = male).

According to the random-effects model, female patients (b = − 0.62; SE = 0.27; *p* = 0.021) were significantly at a lower risk for subsidence over time (Fig. 6). A significant trend towards a higher risk for subsidence was also observed in patients with a high BMI (b = 0.06; SE: 0.03; *p* = 0.064). Age at the time of surgery (*p* = 0.644), stem size (*p* = 0.139), DORR types (A vs. B: *p* = 0.658; A vs. C: *p* = 0.506), and CCD-groups (< 125° vs. 125–135°: *p* = 0.484; < 125° vs. > 135°: *p* = 0.234) did not significantly alter the amount of subsidence over time. In the multivariate model—including age at the time of surgery, sex, and BMI—female sex remained a positive prognostic factor in reducing subsidence over time (Table 2).

**Table 2:** Multivariate random effects model on the course of subsidence.

	b	(SE)	z	*P* value	95% Conf. interval
Time (month)	0.05	0.00	19.3	< **0.001**	0.05; 0.06
Age at surgery	0.01	0.02	0.46	0.644	− 0.03; 0.04
BMI	0.04	0.03	1.45	0.148	− 0.02; 0.10
Gender (female)	− 0.55	0.28	− 1.99	**0.046**	− 1.08; − 0.01
Constant	− 0.78	1.36	− 0.57	0.568	− 3.45; 1.89

Significant values are in [bold].

*BMI* Body mass index, *SE* Standard error.

## Discussion

The present study analyzed the migration pattern of a novel, mainly metaphyseal-anchoring, calcar-guided femoral short stem (ANA.NOVA proxy; ImplanTec, Moedling, Austria) in a 3-year follow-up study, and the influence of patient-related attributes and stem size on axial subsidence. To the best of the authors knowledge, this is the first study to investigate the migration behavior of this short-stem with EBRA-FCA.

After a follow-up of 36 months, moderate subsidence of 2.00 mm with a pronounced subsidence within the first year after surgery was observed. While no significant influence regarding patient age, stem size, DORR types, and CCD groups, female gender was associated with a lower at risk for subsidence. Furthermore, patients with high BMI tended to have a higher risk for subsidence.

It has been described that early stem subsidence after cementless THA correlates with aseptic loosening of the femoral component, being the most common cause for implant failure^28,29,44,45^. Axial migration can be assessed using EBRA-FCA, with a reported specificity of 100% and sensitivity of 78% regarding detection of migration of more than 1 mm^30,31^. Therefore, although RSA is considered the gold standard for migration analysis, EBRA-FCA is a valid tool to assess the subsidence of femoral stems^26,31^.

In several previous studies, short stems’ migration patterns have been assessed with EBRA-FCA^36,46,47^. The Metha (Aesculap Braun, Germany) short stem showed a mean axial migration of 1.96 mm 1 year after THA, and the Nanos stem (Smith & Nephew GmbH, Marl, Germany) an axial migration of 2.04 mm 1 year postoperatively^48^. Unlike Brinkmann et al., Schmidutz et al.^26^ showed a mean subsidence of 0.70 mm 2.7 years after THA with the Metha stem. While the Optimys stem (Mathys Ltd., Bettlach, Switzerland) reached mean subsidence of 1.39 mm at 2 years postoperatively^40^, Schaer et al.^47^, using the same stem, reported a mean subsidence of 1.71 mm. Compared to our findings, average subsidence of 1.55 mm at 2 years postoperatively reached the threshold for a subsidence considered as a risk factor for aseptic loosening and thus earlier implant failure, as described by Krismer et al.^31^. This may give the impression that the ANA.NOVA proxy underperforms in relation to the above-mentioned short stems. However, looking at complication and revision rates, our results are comparable to previously reported ones. Moreover, no association between moderate early subsidence and mid-term aseptic implant loosening requiring surgical revision was herein obsereved. In addition, it has to be noted that Krismer et al.^31^ included cemented and uncemented conventional femoral stems, eventually restricting comparability to cementless short stems only. Compared with the cut-off value of 2.7 mm at 2 years after surgery based on a cementless conventional stem (CLS Spotorno stem: Zimmer Inc, Warsaw, IN, USA) for predicting late aseptic failure with a specificity of 99% and a sensitivity of 56% as described by Streit et al.^29^, the average subsidence of the ANA.NOVA proxy did not surpass the threshold. This might indicate that thresholds for subsidence established in the past are not necessarily applicable to all short stem designs available on the market. Therefore, it seems that individual cut-off values for each design should be established to predict risk for later aseptic loosening adequately^25,27^. However, Streit et al.^29^ additionally stated that most of failed femoral stems in their study showed continuous subsidence after the second year. Contrary to their findings, another study^39^ investigating the Fitmore stem (Zimmer, Warsaw, Indiana, USA) showed that all short stems “at risk” 2 years after surgery were classified as “stable” 5 years postoperatively, again indicating that conventional stems and short stems show different migrations patterns. Owing to the 3-year follow-up, it can herein only assumed that the ANA.NOVA proxy provides satisfactory long-term durability based on the knowledge of how other short stems behave after initially pronounced axial migration.

The current EBRA-FCA revealed average subsidence of 0.25 mm and 2.00 mm after 6 weeks and 3 years, respectively, compared to immediate postoperative measurement. Notably, the median subsidence was higher within the first year than within the second or third year, suggesting that stems stabilize over time, in line with the previously described “settling effect”^25,26,35,36,39^. This is in accordance with the results of a recent study conducted by Dammerer et al. using the Accolade II by Stryker (Stryker, Kalamazoo, MI, USA)^32^. In their study, the main subsidence was found during the first postoperative year, whereby a reduction of the mean monthly migration of 0.11 mm 6 months after surgery to less than 0.02 mm after 24 months could be observed^32^. Not as pronounced as in the study by Dammerer et al.^32^, however, we likewise observed a reduction in the migration progression from year to year (1.20 mm vs. 0.30 mm vs. 0.25 mm). This seems to be a characteristic migration behavior of short stems, supporting the growing assumption that femoral short stem designs could provide enough durability via secondary fixation after an initial progressive migration during the early phase after surgery^25,26,35,36^. This migration pattern can be explained by the tapered and curved design, leading to a wedging in the proximal femur and impaction of trabecular bone^19,25,49^.

In the current study, stem subsidence did not significantly correlate with patient’s age at the time of surgery, DORR types, or CCD groups, being in line with findings of other studies^25,32,47,50^. However, a non-significant trend towards increased subsidence with higher BMI was found, confirming earlier reports^25,35,40^. Kutzner et al.^25,51^ showed that high body weight (i.e. > 75 kg) is associated with an increased subsidence 2 years postoperatively. However, when adjusted for sex and gender, weight does not appear to influence subsidence. Stihsen et al.^52^ investigated the Vision-2000 stem (Depuy Orthopaedics Inc., Warsaw, Indiana, USA) and found that body weight > 75 kg significantly affects subsidence at 2-year follow-up. As body weight seems to be of greater significance than BMI regarding subsidence in femoral short stems, indication for these implants may be made more cautious in heavy patients^25^.

Influence of gender on short stem implant survival has been reported controversially in the literature^25,32,52^. While some studies^40,52^ reported male gender being at higher risk for subsidence, another study^32^ could not find a significant difference. To date, it remains unclear whether increased rate of subsidence reported in males is related to increased weight or activity levels^53^. Based on our results, female gender significantly correlated with a lower risk for subsidence over time. Furthermore, female gender prevailed as a positive predictive parameter in subsidence over time, irrespective of patient’s age or BMI. Influence of gender might again highlight the potentially substantial effect of body weight on increased short stem subsidence, as seen particularly in male and heavy-weight patients^40^. Therefore, in active male and obese patients, the indication for a short stem should be more rigorous, bearing the higher likelihood for migration in mind.

Schaer et al.^47^ were the first to provide data with regard to a correlation of stem size and subsidence investigating the Optimys stem. They reported—in women only—significantly higher subsidence 5 years after surgery with increasing stem sizes (size ≥ 6: 2.97 mm vs. size < 6: 1.48 mm). They attributed this difference to the fact that the surgeon probably did not chose a larger stem owing to the fear of intraoperative periprosthetic fractures^47^. As a result, optimal press-fit fixation may not have been achieved, leading to higher subsidence in these patients. In accordance with the present findings, Kaipel et al. reported that size of the Nanos stem did not affect distal migration. Furthermore, a recent study emphasized the importance of sufficient contact of short stems with the lateral femoral cortex^54^. The cited article reported a significantly increased axial subsidence 5 years after surgery in case the Optimys stem did not touch the lateral femoral cortex (2.07 mm vs. 1.03 mm [stems touching femoral cortex]). This suggests that proper stem size and adequate press-fit are two further important factors regarding migration.

However, this study has to be interpreted in the light of its limitations. First, the 3-year follow-up is relatively short, and the number of included patients limited. Therefore, the impact of migration on potential future aseptic loosening and implant failure could not be determined. Furthermore, only axial subsidence was analyzed, whereas rotation and tilting were not assessed. Third, RSA was not used, albeit being considered the gold-standard to measure migration. Yet, EBRA-FCA is an established and valid method for migration analysis, and can be considered superior to RSA as being non-invasive^30,31^. Moreover, a calcar-guided, “partial collum”-preserving short stem was analyzed, wherefore the results obtained may not be entirely comparable to other short-stem designs.

During the study period, the detected subsidence did not lead to stem loosening, instability, dislocation, or revision surgery in any patient. Nevertheless, long-term results will be necessary to determine the impact of early migration on the current short-stem’s survival.

## Conclusion

Although moderate distal migration of a new short stem (ANA.NOVA proxy) was radiographically detectable with EBRA-FCA—especially within the first year—no correlation between migration and revision 3 years postoperatively was found. Thus, traceable axial subsidence in short stems may be interpreted differently from conventional stems. Furthermore, stem subsidence was higher in males and per tendency also obese patients. Nevertheless, additional long-term studies are needed to assess the impact of early subsidence on implant survival.

## Data Availability

The datasets used and analyzed during the current study are available from the corresponding author upon reasonable request.

## Abbreviations

EBRA-FCA: Einzel-Bild-Roentgen Analyse, Femoral Component Analyse
THA: Total hip arthroplasty
BMI: Body mass index
a.p.: Anteroposterior
IQR: Interquartile range
SE: Standard error
CCD: Caput collum diaphyseal angle
RSA: Radio stereometric analysis
